# Therapeutic relationship in mental health nursing: A scoping review

**DOI:** 10.1192/j.eurpsy.2021.1935

**Published:** 2021-08-13

**Authors:** C. Laranjeira

**Affiliations:** Citechcare, Polytechnic of Leiria, Leiria, Portugal

**Keywords:** mental health nursing, therapeutic relationship, scoping review, caring

## Abstract

**Introduction:**

In mental health nursing, the therapeutic relationship is central to the care process, since the restoration of the balance of the person in mental suffering relies on significant interpersonal relationships.

**Objectives:**

This scoping review aims to map which personal qualities of the nurse favor the therapeutic relationship in mental health nursing.

**Methods:**

A question was formulated according to the PICo method: What are the nurse’s personal qualities that benefit the therapeutic relationship with the patient in mental health settings? For the selection of studies were used the following databases: Cochrane Database of Systematic Reviews; CINAHL; MEDLINE. A survey was carried out, with the following Boolean conjugation (nurse AND patient) and (personal AND qualities) and (mental AND health) and (therapeutic relation OR relation*). The limit applied to this research was the full text.

**Results:**

A total of 12 studies were analyzed. These are predominantly qualitative with different methodological approaches. The nurse’s personal attributes or qualities imply not making judgments, be patient, be open and genuine. It was also evidenced the importance of the professional and personal dimensions in the therapeutic relationship.

**Conclusions:**

In all studies, it was clear that the therapeutic relationship is influenced by attributes of the professional dimension that are linked, mainly, with the theoretical domain, technical knowledge and by attributes of the personal dimension that are related with the professional’s personal qualities or characteristics. The strategies used for the development of the therapeutic relationship imply the involvement of the person nurse, using this to elements of the personal and social sphere.

**Disclosure:**

No significant relationships.

